# Short-Term Fluctuation of Subjective Age and its Correlates: An Ecological Momentary Assessment of Older Adults

**DOI:** 10.1093/geroni/igab046.1108

**Published:** 2021-12-17

**Authors:** Anna Kornadt, Theresa Pauly, Denis Gerstorf, Ute Kunzmann, Oliver Schilling, David Weiss, Anna Luecke, Hans-Werner Wahl

**Affiliations:** 1 University of Luxembourg, Esch-sur-Alzette, Luxembourg; 2 University of Zurich, University of Zurich, Zurich, Switzerland; 3 Humboldt University of Berlin, Humboldt University of Berlin, Brandenburg, Germany; 4 Leipzig University, Leipzig, Sachsen, Germany; 5 University of Heidelberg, Heidelberg, Baden-Wurttemberg, Germany; 6 Heidelberg University, Heidelberg, Baden-Wurttemberg, Germany

## Abstract

We examined short-term fluctuations of subjective age with data obtained from 123 young-old (Mage = 67.19 years) and 47 old-old adults (Mage = 86.59 years) who reported their momentary subjective age six times a day over seven consecutive days as they were going about their everyday lives. Participants felt younger on a large majority of occasions, and 25% of the total variability in subjective age could be attributed to within-person variation. Those with younger trait subjective ages exhibited larger moment-to-moment variation, while chronological age did not impact variability. Furthermore, we investigated relationships between within-day fluctuations of subjective age and daily cortisol fluctuations. Our findings extend the literature on subjective age by showing that how old people feel can vary on a momentary basis, that state and trait components of subjective age are related, and that fluctuations in subjective age are related to biomarkers of stress.

